# King Edward's Hospital Fund for London

**Published:** 1906-04-14

**Authors:** 


### HOSPITAL ADMINISTRATION.

### CONSTRUCTION AND ECONOMICS.

### KING EDWARD'S HOSPITAL FUND FOR LONDON.

THE ANNUAL MEETING.

The annual meeting of the General Council of King Edward's Hospital Fund for London was held on the 6th inst. at 15 Portman Square.

The Duke of Fife, as Acting President of the Fund during the absence of the Prince of Wales, presided.

Those present were: Lord Bessborough, Lord Rothschild, the President of the Hospital Sunday and Hospital Saturday Funds (the Lord Mayor), the Chairman of the County Council (Mr. Evan Spicer), the Governor of the Bank of England (Mr. A. F. Wallace), the President of the Royal College of Physicians (Sir R. Douglas Powell), the President of the Royal College of Surgeons (Mr. John Tweedy), Sir Savile Crossley, Sir John Aird, Sir W. S. Church, Sir W. J. Collins, M.P., Sir John Craggs, Mr. Frederick M. Fry, and Mr. Hugh C. Smith.

Lord Rothschild, the Hon Treasurer, in submitting the accounts, said that the accounts of receipts and expenditure for the year ended December 31, 1905, were of a very simple and satisfactory nature. The Fund received in the year 1905 no less a sum than £361,068. Of this sum of £361,068, £237,206 represented money which would be added to their capital. A satisfactory feature in connection with the accounts is that the total expenditure for carrying on the work of the Fund does not amount to ¾ per cent. on the amount collected. As far as the balance-sheet is concerned, there is at the present time a capital sum of very nearly a million sterling.

THE APATHY OF LONDONERS.

The Duke of Fife, after referring to the great work which the King's Fund has accomplished during the nine years of its existence, called attention to the passive attitude which the general public adopt towards the maintenance of the London hospitals. The great majority of the public, he said, do not subscribe at all, and the plain truth is that the London hospitals are maintained by a small number of charitable people. The needs and necessities of the hospitals have grown enormously in the last twenty years, and if it were not for doubtful finance, sensational appeals, and every form of begging, many of them would be obliged to close their doors.

THE LEAGUE OF MERCY.

In saying this he did not in any way wish to ignore the excellent work done by the League of Mercy. But, though the League collects large sums from a great many different donors, there must still be an enormous number of people in this vast city who could afford to give their guineas, or even more, but who render no assistance to the hospitals.

LETTER FROM THE PRINCE OF WALES.

The Duke of Fife then read the following letter which he had received from the Prince of Wales:—

"Prince of Wales's Camp, India, February 19, 1906.

"I regret that my absence from England makes it impossible for me to preside over the annual meeting of the General Council without causing an undue delay in the issue of the annual report and statement of accounts of the Fund. I should be sorry to thus prevent the subscribers receiving the earliest possible information as to the work of the past year and the general position of the Fund. That information will, I think, be considered as very satisfactory. We have, as I stated in my last letter, now secured a permanent income from investments of £50,000. The steady increase in the annual subscriptions and the large sums received in legacies strengthen me in the belief that the Fund is trusted by the public and that its work meets with their approval.

"It is especially satisfactory to know that no less than £22,500 was given to the Fund by executors who were left a discretion, while a further amount of £5,000 was sanctioned by a legal distribution of a sum bequeathed by the late Mr. T. J. Bell.

"In addition to the usual work of visiting every hospital which receives our grants, and the careful and prolonged labours of the Distribution Committee, based on the visitors' reports, we have this year made exhaustive reports on the financial relations of London hospitals and their medical schools, and on hospital accounts. I have already expressed my thanks to those gentlemen responsible for them. It is to be hoped that the steps which I am glad to see are being taken by most of the hospitals will result in the difficulty regarding medical schools being shortly removed. I trust that with the co-operation of the hospital officials themselves improvement in the system of keeping accounts will be beneficial to all concerned.

"The Fund continues to study with careful attention the question of economy in maintenance, to which I referred at length in 1904. I am convinced that co-operation in the circulation of the information obtained from the various hospitals cannot but be helpful to those responsible for their administration.

"The difference in the cost per bed for the same items for hospitals in the same class is still very striking. I hope that our statistical report, which has already produced valuable practical results, will be the means of still further saving. Several of the hospitals have shown their appreciation of this effort, and have recognised its great service to them in re-arranging their management on a more economical basis, and I am glad to know that our work has already borne good fruit.

"I must take this opportunity of acknowledging the great kindness of Mr. George Herring in reserving to the King's Fund the repayment of the munificent sum he is placing at the disposal of the Salvation Army for a farm colonisation scheme.

"In conclusion, I wish to thank the members of the Council and of the various committees, as well as the honorary officials, for their services to the Fund during the past year. I have also to thank Mr. Danvers Power, as well as all the hospital officials and others who have assisted in the production of our most valuable statistical report."

The Lord Mayor seconded the adoption of the accounts, which was supported by Lord Bessborough, and carried unanimously.

REPORT OF THE GENERAL COUNCIL.

Sir Savile Crossley read the draft report of the General Council for the year 1905.

The sums received during 1905, in addition to interest from investments (£38,874 8s. 10d.), were as follows:—Donations, £7,811 16s. 7d.; annual subscriptions, £36,023 14s. 11d.; from the League of Mercy, £15,000. The total income of the Fund from general sources amounted to £123,862 5s. 4d. There was received £237,205 14s. 7d. on capital account. The year 1905 was marked by the accomplishment of what had been the immediate object of the Fund for some years—namely, a permanent income of £50,000 per annum. This gratifying result was due to a number of munificent gifts to capital received during the year. To Lord Mount Stephen, who gave £200,000 to the Fund in 1902, the grateful thanks of all connected with the Fund are due for the further munificent donation of £200,000.

Every eligible hospital which applied for a grant was inspected and reported upon. The Council have continued to urge on some of the smaller special hospitals the desirability of amalgamation, and, though they have no definite announcement to make this year in addition to what has been stated on previous occasions, they have hopes that their efforts will bear fruit in the near future. The second statistical report was issued last summer, and dealt with 36 hospitals, as against 16 in the previous year. The Council have received gratifying testimony from several of the hospitals as to the reduction in expenditure which has been found possible in consequence of the information contained in the report.

The Medical Schools.

Although on the occasion of the annual meeting last year His Royal Highness, the President, was able to thank the Medical Schools Committee for the services they rendered to the Fund in inquiring into the financial relations between hospitals and their medical schools, their work was actually concluded during the last financial year. The Council take this opportunity of thanking Sir Edward Fry (the chairman), Lord Welby, and the Bishop of Stepney for their report, which was adopted, and which has been acted upon. The difficulty in regard to medical schools, has, it is hoped, been now nearly overcome. While the Council regarded the original understanding between the Fund and its subscribers to be that no part of the money contributed to the Fund should go to medical laboratories, and, as being in the nature of a trust, they fully adhere to the view that the public look to the voluntary hospitals for the means of medical education and the advancement of medical science. It has, however, been shown that among the subscribers to hospitals there are quite sufficient who are willing that their subscriptions should, if necessary, be employed for the purpose of medical education. On the other hand, there are others who, while wishing to help towards the immediate relief of the sick poor, are not willing to contribute for any other purpose. The Fund has also to bear in mind that its permanent income of £50,000 per annum is probably largely derived from capital gifts made in reliance on the original constitution of the Fund. The Council see no reason to object to the subscribers to hospitals direct ear-marking their contributions either for a hospital or a medical school, as this is a matter for their own consideration and is entirely distinct from grants made by the King's Fund out of a sum which is the result of a great number of contributions invited for the direct relief of the sick poor. The Council are glad to know that it is probable that, though the work of placing this matter on a proper basis may have caused some inconvenience at the outset, in the long run the schools will be placed in a more suitable position than they have occupied in the past, and that benefit to medical education will be the result.

Hospital Accounts.

A wish having been expressed by many hospital authorities that the uniform system of hospital accounts should be revised, for the purpose of enabling more accurate comparisons of the expenditure between different hospitals to be made, His Royal Highness, the President, appointed Mr. J. G. Griffiths, formerly the head of the firm of Deloitte, Plender, Griffiths and Co., the honorary auditors of the Fund, to inquire into and report upon the matter. The Council desire to express their thanks to Mr. Griffiths for a most valuable report, which has been laid before the Metropolitan Hospital Sunday Fund and the Hospital Saturday Fund. Steps are now being taken which will enable the views of the hospital officials to be laid before the three funds in order that whatever changes are eventually adopted may, as far as possible, meet the views of all concerned. The attention of the Council has been called to the increasing number of proposals made to the Fund in connection with schemes to be carried on for profit, a part of which is offered to the Fund. In some cases these offers are made with the sole intention of benefiting the Fund, but in others they amount to little more than offering a commission for the use of the Fund's name. The Council have thought it better to make a rule to the effect that, while the Fund is of course grateful for all assistance given to it, it cannot officially allow the use of its name in connection with schemes for raising money in any form other than direct contributions to the Fund itself. In conclusion, the Council again rely upon the kindly co-operation of the Press in obtaining for the work of King Edward's Hospital Fund for London yet wider publicity and support.

The Duke of Fife moved the adoption of the report, which was seconded by Lord Rothschild, supported by Sir John Aird, and carried unanimously.

The President of the Royal College of Physicians (Sir R. Douglas Powell) proposed, and the Governor of the Bank of England (Mr. A. F. Wallace) seconded, a vote of thanks to the President, which was carried unanimously, and which the Duke of Fife acknowledged.

The proceedings then terminated.

